# Clinical characteristics and long-term prognosis of relapsing anti-*N*-methyl-d-aspartate receptor encephalitis: a retrospective, multicenter, self-controlled study

**DOI:** 10.1007/s10072-020-04482-7

**Published:** 2020-06-29

**Authors:** Wei Zeng, Liming Cao, Jinou Zheng, Lu Yu

**Affiliations:** 1 Department of Neurology, Liuzhou People’s Hospital, Liuzhou, China; 2 Department of Neurology, The 3rd Affiliated Hospital of Shenzhen University, Shenzhen, China; 3 Department of Neurology, First Affiliated Hospital of Guangxi Medical University, Nanning, China

**Keywords:** Anti-*N*-methyl-d-aspartate receptor encephalitis, Relapse, Epilepsy, Psychiatric, Cognitive dysfunction, Long-term prognosis

## Abstract

**Objective:**

To analyze the clinical profile and long-term prognosis of relapsing anti-*N*-methyl-d-aspartate receptor (NMDAR) encephalitis.

**Method:**

This is a retrospective, multicenter, self-controlled study of 10 patients with relapsing anti-NMDAR encephalitis. Relapse was defined as new psychiatric or neurologic syndrome unexplainable by other causes that improved after immunotherapy.

**Results:**

The main symptoms at first onset and relapse included psychiatric symptoms, cognitive impairment, speech dysfunction, seizures, consciousness disturbance, movement disorders, central hypoventilation, and autonomic dysfunction. There were significantly fewer seizures and consciousness disturbances at relapse. At the first onset, the antibody positivity rate was significantly higher in the cerebrospinal fluid (CSF) than in the serum, and abnormal electroencephalograms results were noted in all patients. The relapse rate was 12.2%. After first-onset discharge, the duration of medication intake was 3.10 ± 2.69 months; the relapse time was 18.3 ± 16.5 months. The Modified Rankin Scale (MRS) score at relapse was significantly lower than that at the first onset. The MRS scores at relapse and first onset after immunotherapy were significantly lower than those before immunotherapy. At follow-up, the average duration of antiepileptic drug (AED) intake was < 1 year; the relapse rate was low.

**Conclusions:**

Patients have fewer symptoms and better quality of life at relapse than at the first onset. Active immunotherapy can significantly improve the quality of life during first onset and relapse. Encephalitis antibody testing in the CSF is preferred at first onset and relapse. Increasing antibody titers suggest clinical relapse. Prematurely stopping immunotherapy may lead to relapse, but prolonged AED intake is unnecessary.

## Introduction

Anti-*N*-methyl-d-aspartate receptor (anti-NMDAR) encephalitis is a rapidly progressing encephalopathy characterized by abnormal behavior, cognitive dysfunction, speech dysfunction, seizures, movement disorders, consciousness disturbance, autonomic dysfunction, and central hypoventilation [1]. It accounts for approximately 80% of all autoimmune encephalitis cases [2, 3]. Specific reports on relapsing anti-NMDAR encephalitis are lacking, and the clinical characteristics and prognosis of relapse are not well established; therefore, diagnosis is mainly based on symptomatology, antibody detection, and effect of immunotherapy. This study aimed to analyze the clinical data and long-term follow-up of patients with first-onset and relapsing anti-NMDAR encephalitis to improve the diagnosis and treatment, as well as prevent relapse.

## Material and methods

This retrospective, multicenter, self-controlled study analyzed 82 patients with anti-NMDAR encephalitis from February 2013 to September 2018. The diagnostic criteria of anti-NMDAR encephalitis [4] are rapid onset (course < 3 months) of at least one of the following symptoms: abnormal behavior (psychiatric symptoms) or cognitive dysfunction, speech dysfunction (uninterruptible imperative language, being quiet), epileptic seizure, movement disorders (dyskinesia, rigidity, or postural difficulties), decreased level of consciousness, autonomic dysfunction, or central hypoventilation; positive cerebrospinal fluid (CSF) anti-NMDAR antibody test; and no other possible causes, such as viral encephalitis and herpes simplex encephalitis. Relapsing anti-NMDAR encephalitis [5] was defined as any new psychiatric or neurologic syndrome that cannot be explained by other causes and that improved after immunotherapy or, less frequently, spontaneously. The follow-up duration was 12–55 months after the first onset. Patients with no relapse were excluded. Finally, 10 patients with relapse (9 patients at the First Affiliated Hospital of Guangxi Medical University and 1 patient at Hechi People’s Hospital; 6 males and 4 females) with a mean age of 25.6 ± 14.1 years (range, 8–50 years) at the first onset were included.

### Examinations

The clinical symptoms, CSF/serum antibody, electroencephalogram (EEG), and magnetic resonance imaging (MRI) results of 10 patients with relapsing anti-NMDAR encephalitis were analyzed. The Modified Rankin Scale (MRS) was used to assess the neurologic status and quality of life (QOL).

Auxiliary examinations included indirect immunofluorescence analysis to test for anti-NMDAR antibody (Jinyu Inspection Co., Ltd., Guangzhou, GD, China); video EEG (Thermo Nicolet Corporation Guangzhou Branch, Guangzhou, GD, China) for ≥ 3 h; 1.5/3.0-T brain MRI (General Electric Company, Boston, MA, USA), including T1- and T2-weighed imaging, fluid attenuation inversion recovery, and enhancement sequences; tumor screening, including tests for serum tumor markers such as ferritin, carcinoembryonic antigen, carbohydrate antigen (CA) 125, CA153, CA199, squamous cell carcinoma antigen, and α-fetoprotein test; chest computed tomography (CT) (Siemens, Berlin, Germany); and gynecologic color Doppler ultrasound.

The EEG results were categorized as follows [6]: normal, dominant α-rhythm, and a few occasional decentralized θ wave; slight abnormality, α-wave dysrhythmia, decreased frequency, a high-amplitude β wave exceeding 50 μV or high-amplitude θ wave, and a medium-amplitude σ wave; moderate abnormality, frequency of α wave is low or dominant θ-wave activity, cluster or group appearance of a middle-amplitude σ-wave, and asymmetrical bilateral electrical brain activity; severe abnormality, widespread θ- or σ-wave or even spikes, sharp wave, or spike/sharp and slow-wave complexes.

### Immunotherapy

The first-line treatment included pulsed methylprednisolone therapy (1 g for 5 days, 0.5 g for 3 days, 0.25 g for 2 days, and 0.125 g for 1 day; Pfizer Manufacturing, Belgium) and prednisone (Zhejiang Xianju Pharmaceutical Co., Ltd., Zhejiang, China) at 1 mg/kg/day substituted and plasma exchange and intravenous immunoglobulin (0.4 g/kg/day for 5 days; Chengdu Rongsheng Pharmaceutical Co., Ltd., Chengdu, China). The second-line treatment included administration of azathioprine (100 mg/day; Shanghai Xinyi Pharmaceutical Co., Ltd., Shanghai, China) and metoclopramide (1 g/day; Hangzhou Zhongmei Huadong Pharmaceutical Co., Ltd., Hangzhou, China).

### Statistical analyses

The mode of onset, main clinical manifestations, auxiliary examination results, treatment strategies, and prognosis for each patient were retrospectively analyzed using SPSS version 21.0 (IBM Corp., Armonk, NY, USA). The measured data exhibited normal distribution and were expressed as mean ± standard deviation. Student’s *t* test was used to compare between the MRS scores before and after treatment for paired data. The clinical symptoms and detection of antibody were compared using the exact probability method.

## Results

The rate of relapse was 12.2% (10/82). The MRS score at relapse was significantly better than that at first onset. Patients were less likely to be admitted to the intensive care unit (ICU) at relapse than at first onset.

### Clinical manifestations

The incidence of prodromal symptoms was significantly higher at first onset than at relapse. The main symptoms at both time-points were psychiatric symptoms, cognitive impairment, speech dysfunction, seizures, consciousness disturbance, movement disorders, central hypoventilation, and autonomic dysfunction. Patients had significantly fewer episodes of epileptic seizures and consciousness disturbance at relapse than at first onset. There was no significant difference in the incidence of the other symptoms between the two groups (*P* < 0.05; Table 1).

**Table 1:** Clinical features, auxiliary examinations, treatments, and follow-up of patients with first-onset and relapsing anti-NMDAR encephalitis

	Case 1	Case 2	Case 3	Case 4	Case 5	Case 6	Case 7	Case 8	Case 9	Case 10	First onset (%)	Relapse (%)	*P*/*χ*^2^
Sex/age, year	F/18	M/45	M/31	M/20	M/8	F/50	M/14	M/17	F/17	M/36			
Prodromal symptoms^(1/2)^	+/+	−/−	+/−	−/−	+/+	−/−	−/−	+/−	+/−	+/−	6 (60)	2 (20)	0.085
Psychiatric^(1/2)^	+/+	+/+	+/−	+/+	−/+	+/+	+/−	+/+	+/−	+/−	9(90)	6 (60)	0.152
Cognitive impairment^(1/2)^	+/+	+/+	+/−	+/+	+/+	+/+	+/+	+/+	+/−	+/−	10 (100)	7 (70)	0.105
Speech dysfunction^(1/2)^	+/+	−/−	−/+	−/−	+/+	−/−	−/−	−/−	−/−	+/−	3 (30)	3 (30)	0.686
Seizures^(1/2)^	+/−	−/−	+/+	+/−	+/−	−/−	+/+	+/−	+/+	+/−	8 (80)	3(30)	0.035a
Consciousness disturbance	+/−	−/−	+/−	+/−	−/−	+/−	+/−	−/−	+/−	−/+	6 (60)	1(10)	0.029b
Movement disorders^(1/2)^	+/−	−/−	−/+	−/−	+/−	+/+	+/−	−/−	+/−	−/−	5(50)	2 (20)	0.175
Central hypoventilation^(1/2)^	−/−	−/−	+/−	+/−	+/−	−/−	−/−	−/−	−/−	−/−	3 (30)	0 (0)	0.105
Autonomic dysfunction^(1/2)^	+/+	+/+	−/−	+/−	+/−	+/−	−/−	−/−	−/−	+/−	6 (60)	2 (20)	0.085
ICU admission^(1/2)^	−/−	−/−	−/−	+/−	+/−	−/−	−/−	−/−	−/−	−/−	2 (20)	0 (0)	0.237
CSF WBC (1/2), × 106/L	−/30	−/−	−/20	20/−	−/−	55/−	−/−	−/−	37/−	−/−	3/7 (30)	2/8 (20)	0.500
CSF protein (1/2), ×mg/L	−/−	−/−	453/543	−/−	−/−	−/−	−/−	−/−	570/−	542.8/592.4	2/8 (20)	2/8 (20)	0.709
CSF/serum antibody^1^	1:1/1:10	1:10/N	1:10/N	1:1/N	1:32/1:320	1:32/1:32	1:32/N	1:10/1:1	1:10/1:10	1:1/N	10 (100)	5 (50)	0.016c
CSF/serum antibody^2^	Not done	1:10/1:100	1:32/1:100	1:3.2/N	1:32/1:32	1:1/1:100	1:10/1:32	1:3.2/1:10	Not done	1:3.2/N	8/8 (100)	6/8 (75)	0.233
EEG^1^	Moderate abnormality	Not done	Moderate abnormality	Not done	Mild to moderate abnormality	Severe abnormality	Moderate to severe abnormality	Moderate abnormality	Moderate to severe abnormality	Mild to moderate abnormality	8/8 (moderate to severe abnormality, 100)		0.500
EEG^2^	Not done	Moderate abnormality	Severe abnormality	Moderate abnormality	Moderate abnormality	Mild abnormality	Moderate abnormality	Moderate 2abnormality	Severe abnormality	Not done		7 /8 (87.5)	
Brain MRI^1^	–	White matter demyelination	Lobes, basal ganglia	–	–	–	–	–	–	Midbrain, and lobe	3/10 (abnormality, 30)		0.564
Brain MRI^2^	–	White matter demyelination	Lobes, basal ganglia	–	–	–	–	–	Hippocampus, lobe	Brainstem, basal ganglia, lobes, etc.		4/10 (40)	
MRS of pretreatment and posttreatment^1^	5/2	3/2	5/2	5/0	5/4	4/1	5/1	3/0	5/2	5/2	4.50 ± 0.85d, pretreatment	1.60 ± 1.17d, posttreatment	0.000d
MRS of pretreatment and posttreatment^2^	3/1	3/2	4/2	2/0	3/2	3/1	3/1	2/0	3/1	3/2	2.90 ± 0.57e	1.2 ± 0.79e	0.000e
Immunotherapy^1^	MP	MP+ HGG+AZA	MP	MP+HGG+PE	MP+HGG 2 times	MP+PE 3 times	MP+AZA+HGG	MP	MP+HGG+PE 4 times	MP+AZA	7/10f (≥ 2 immunotherapies)	6/10g (≥ 2 first- line immunotherapies)	0.500f
Immunotherapy^2^	MP, HGG+AZA	MP+ HGG+AZA	MP+HGG	MP+ MMF	MP+HGG	MP	MP+AZA	MP+MMF	MP+MMF	MP	8/10f (≥ 2 immunotherapies)	4/10g	0.328g
Postdischarge immunotherapy^1^, months	PED > 2	PED+AZA > 10	PED 2-3	PED+AZA 3	PED > 3	PED 1	PED+AZA3	No treatment	PED 4	PED+AZA 2	3.10 ± 2.69h		0.022h
Time to relapse, months	4	18	41	4	4	20	22	52	10	8	18.3 ± 16.5h		
Postdischarge immunotherapy^2^, months	PED+AZA 6	PED+AZA 5	PED 2	PED+MMF 6	PED > 10	PED 5	PED+AZA 4	PED+MMF 3	PED+MMF 12	PED 5			
Follow-up time, months	12	33	43	13	30	25	29	55	26	36			

Results are expressed as first onset/relapse,^1^at first onset; ^2^at relapse

MRS scores are expressed as mean ± standard deviation. +, positive symptom or yes; −, negative symptom or no; N, negative results. The relapse events in this table refer to the second relapse

^a^Number of patients with seizure at first onset vs at relapse

^b^Number of patients with consciousness disturbance at first onset vs at relapse

^c^Number of patients with antibody positivity in the CSF vs the serum antibody positivity

^d^Pretreatment vs posttreatment MRS scores at first onset

^e^Pretreatment vs posttreatment MRS score at relapse

^f^Number of patients with ≥ 2 immunotherapy sessions at first onset vs at relapse

^g^Number of patients with ≥ 2 first-line immunotherapy session at first onset vs at relapse

^h^Duration of medication intake after first-onset discharge vs the relapse time

*AZA*, azathioprine; *CSF*, cerebrospinal fluid; *EEG*, electroencephalogram; *F*, female; *HGG*, human γ-globulin; *ICU*, intensive care unit; *M*, male; *MMF*, mycophenolate mofetil; *MRI*, magnetic resonance imaging; *MP*, methylprednisolone; *MRS*, Modified Rankin Scale; *PED*, prednisone; *PE*, plasma exchange

### Auxiliary inspection

#### Anti-NMDAR antibodies in the CSF/serum and routine CSF analysis

At first onset, the rates of antibody positivity in the CSF and serum were 100% (10/10) and 50% (5/10), respectively (*P* = 0.016). At relapse, the rates of antibody positivity in the CSF and serum were 100% (8/8) and 67% (6/8), respectively (*P* = 0.233). At relapse, 83.3% (5/6) of the patients had higher serum antibody titer than at first onset. Furthermore, at relapse, the CSF antibody titer of 50.0% (4/8) of the patients was higher than that at first onset, and the CSF antibody titer of 25% (2/8) of the patients was equal to that at first onset. At first onset, the CSF pressure increased in five patients; routine CSF examination showed higher white blood cells (mainly lymphocytes, reference range, 0–10 × 10^6^/L) in three patients (Table 1). Biochemical CSF analysis showed increased protein levels (reference range, 150–450 mg/L) in three patients (Table 1) and normal sugar and chloride levels. At relapse, the CSF pressure increased in three patients. Routine CSF examination showed that the number of white blood cells (mainly lymphocytes) increased in two patients. Biochemical CSF analysis showed increased protein levels in two patients (Table 1) and normal sugar and chloride levels. The incidence of CSF abnormality of young patients appeared to be high, but there was no significant difference (*P* > 0.05) between the CSF results in patients aged < 40 years and those aged ≥ 40 years (Table 1).

#### Results of tumor screening

At first onset and relapse, the levels of tumor markers (CA125, CA153, CA199, carcinoembryonic antigen, α-fetoprotein, and serum ferritin) and lung CT, color Doppler ultrasonography of the urinary system, and gynecologic color Doppler ultrasound results were normal; small-cell lung cancer or teratoma was not detected. During follow-up, hemoptysis or other neoplastic symptoms were not found.

#### Electroencephalogram

The incidence of abnormal EEG findings at first onset and relapse was 100%, whereas that of moderate–severe abnormal EEG findings was 100% and 87.5% (*P* = 0.500) at first onset and relapse, respectively. Two patients with severe disease (MRS ≥ 4) and poor prognosis had slow waves and δ waves in EEG.

#### Brain magnetic resonance imaging

**Fig. 1:**
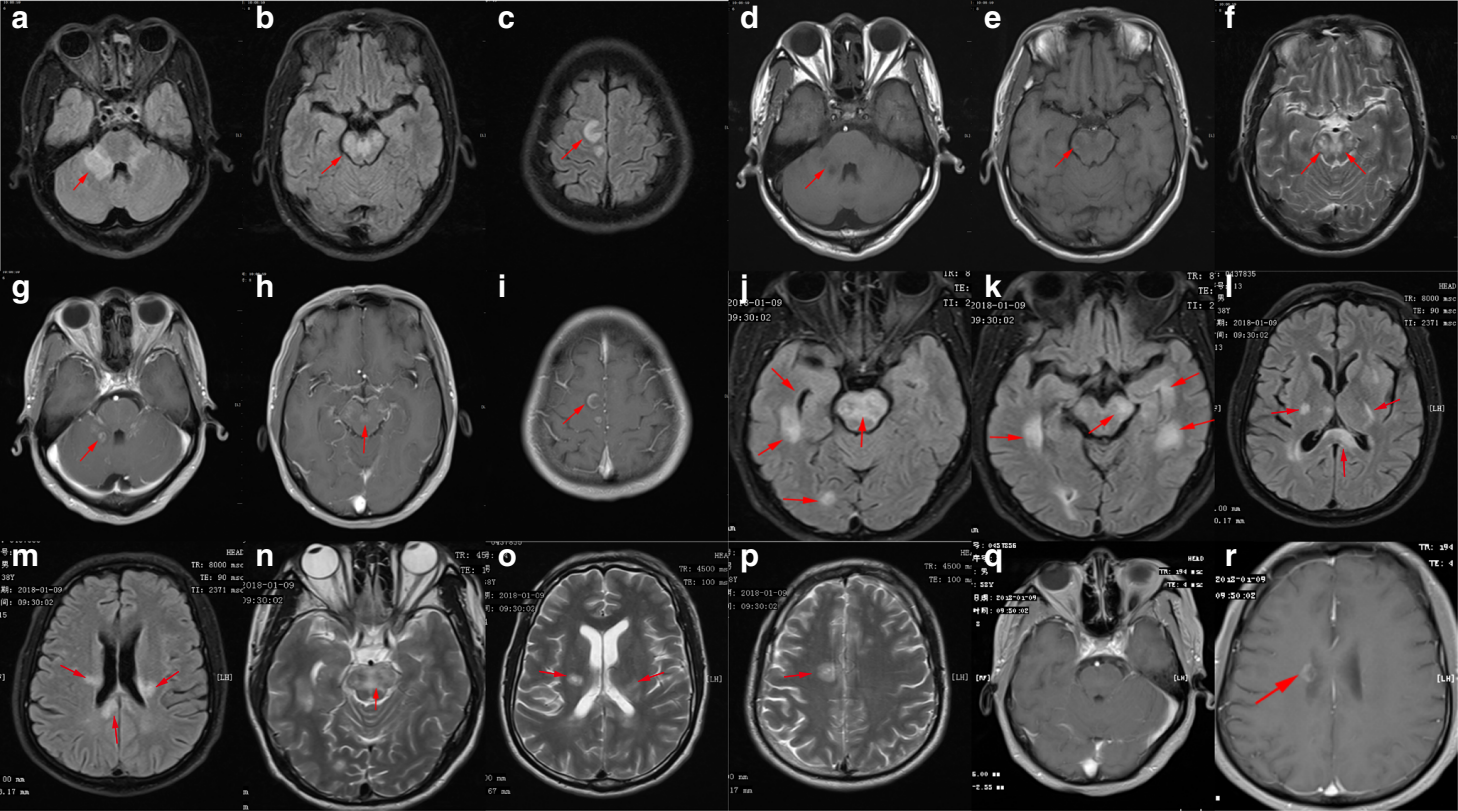
Brain magnetic resonance images of a patient (case 10) with anti-*N*-methyl-d-aspartate receptor encephalitis at first onset (**a**–**i**) and relapse (over 2 years after first onset) (**j**–**r**). Fluid attenuation inversion recovery (FLAIR) sequence showing hyperintense lesions bilaterally in the medi-peduncle (**a**, arrow), most of the midbrain (**b**, arrow), and the local parietal lobe (**c**, arrow). T1-weighted sequence showing low signal intensity in the aforementioned lesions (**d**, **e**, arrow). T2-weighted sequence showing multiple different-sized hyperintense lesions (**f**, arrows). Contrast-enhanced FLAIR sequence showing ring-shaped (**g**, arrow), irregularly shaped (**h**, arrow), and C-shaped (**i**, arrow) lesions in the medi-peduncle, midbrain, and parietal lobe, respectively. FLAIR sequence showing hyperintense lesions in the whole pons (**j**, arrow), occipital lobe (**j**, arrow), whole midbrain (**k**, arrow), left hippocampus (**k**, arrow), bilateral temporal lobes (**k**, arrow), basal ganglia (**l**, arrow), splenium of the corpus callosum (**l**, arrow), and paraventricular regions bilaterally (**m**, arrow). T2-weighted sequence showing irregularly shaped, multiple, different-sized, and circular hyperintense lesions in the pons (**n**, arrow), bilaterally in the basal ganglia (**o**, arrow), and parietal cortex (**p**, arrow), respectively. Lesions are not visible in the pons (**q**), but the C-shaped lesion (**r**, arrow) is visible in the paraventricular region in the contrast-enhanced sequence

The brain MRI scans showed more lesions at relapse than at first onset, but there was no significant difference in the number of patients who had lesions. Brain MRI revealed lesions in the basal ganglia, hippocampus, corpus callosum, and lobes, as well as lesions located in the nonmarginal lobe (Table 1 and Fig. 1). At relapse, the location of the focus was either at the same site as that at the first onset or at another location.

### Treatment and follow-up

Immunotherapy is the primary treatment for anti-NMDAR encephalitis. First-line immunotherapy is the main treatment for first-onset and acute-stage relapsing anti-NMDAR encephalitis, and second-line immunotherapy is added when the effect of the first-line immunotherapy is not sufficient. At first onset and relapse, 70% and 80% of the patients, respectively, were treated with two or more types of immunotherapy (*P* = 0.500), and 60% and 40% of the patients, respectively, were treated with two or more types of first-line immunotherapy.

Regardless of onset, the quality of life (QOL, evaluated using the MRS) of the patients who received active immunotherapy was significantly better than that on admission (both *P* < 0.000; Table 1). After first-onset discharge, the duration of medication intake was 3.10 ± 2.69 months. The relapse time was 18.3 ± 16.5 months after first-onset discharge (*P* = 0.022), and all patients relapsed after discontinuing immunotherapy. Eight patients with epilepsy at first onset were treated with antiepileptic drugs (AEDs; usually two or more). Most patients insisted on taking AEDs for 3–12 months (median 0.5 years). At follow-up, patients with relapsing anti-NMDAR encephalitis had a total epileptic seizure incidence of 3 person-times, which was low.

## Discussion

Relapsing anti-NMDAR encephalitis is commonly observed (15–25%) [5, 7, 8]; however, studies on disease relapse at present are largely overlooked by clinicians. The clinical characteristics and prognosis of relapse are not well established. It is very important to clarify its clinical characteristics to prevent relapse because encephalitis can severely impact work, study, and the overall QOL.

Previous studies [5] have reported that the condition of patients at relapse is better than that at first onset. In this study, the patients’ symptoms and the MRS scores appear to be in line with the previous findings, including the significantly lower incidence of epilepsy and consciousness disturbance at relapse than at first onset (both *P* < 0.05). Some patients may present single neurological or psychiatric symptoms at onset, and other symptoms may not develop until weeks or months after onset. Relapse may present with partial symptoms or with isolated symptoms of the full-blown syndrome. Involuntary movements are more commonly observed among patients with anti-NMDAR encephalitis, who may show abnormal violent movements, including oral-facial involuntary movement, limb tremor, chorea, and even opisthotonos [9]. The patients may have various forms of sleep disorders, including insomnia, rapid eye movement sleep behavior disorder, excessive daytime sleepiness, and sleep–wake cycle disorder. Autonomic instability is usually a late finding. These symptoms usually progress over the course of 1–2 weeks. There are reports that anti-NMDAR encephalitis may affect the brainstem and cerebellum, causing diplopia, ataxia, motor paralysis, and other symptoms [10, 11]. The symptoms of encephalitis vary, and clinicians should pay attention to identifying potential symptoms, which can facilitate early diagnosis and monitoring of curative effects.

In this study, analysis of the CSF pressure and routine and biochemical testing of the CSF at first onset and relapse showed no obvious specificity, and some patients presented changes similar to those noted in virus-like infection, overlapping previous findings [12]. The anti-NMDAR encephalitis antibody is a highly specific diagnostic indicator [13] at first onset and relapse. Our study showed that the rate of antibody positivity in the CSF was 100% at both relapse and first onset and that the patients may test negative for the serum antibody, observations, which are consistent with the results of previous studies [9, 10]. The rate of antibody positivity was higher in the CSF than in the serum, which may be due to the relationship between antibody production and intrathecal synthesis [14]. These findings suggest that encephalitis antibody testing in the CSF is preferred at both first onset and relapse. The detection of anti-NMDAR antibody has a high diagnostic specificity and is more sensitive than the detection of serum antibody. Our study indicated that patients had higher serum and CSF antibody titers at relapse than at first onset (83.3% and 50.0%, respectively). Antibody titers did not predict the therapeutic effect of first-line immunotherapy in cases of anti-NMDAR encephalitis [15]; however, patients were reported to achieve obvious clinical recovery, which was usually accompanied by a decrease of CSF and serum antibody titers [9]. Changes in the antibody levels in the CSF and serum have implications for relapse [9, 16]. Teratomas that contain neural tissue could trigger an immune response resulting in the overproduction of anti-NMDAR antibodies [17], and when these titers are persistent in patients with a prolonged relapsing disease course, the presence of tumors should be considered [18]. Thus, periodic screening of serum and CSF is useful to assess both therapeutic effects and possible relapse.

The MRI results of anti-NMDAR encephalitis are not specific. In our patients, MRI showed abnormal signals in the medial temporal lobe and cingulate gyrus; however, the lesions could exceed the marginal lobes (including the frontal and parietal lobes, with meningeal enhancement, and even absence of lesions; Fig. 1). The lesions found on MRI are helpful for diagnosing encephalitis, and enlargement of the lesions is important for estimating relapse. Herein, MRI showed that there were more lesions at relapse than at first onset (Fig. 1); however, the number of patients with lesions did not significantly differ between those at first onset and relapse (*P* = 0.564). Despite clinical recovery, long-term morphological changes may develop in the brain [19].

The focus of tumor screening should be on ovarian teratoma. Early removal of teratoma is helpful in reducing recurrence. Gynecologic color Doppler ultrasound or pelvic CT is recommended. In this study, all relapse patients tested negative on tumor screening.

At first onset and relapse, the incidence of abnormal EEG findings was high (both 100%), and the incidence of moderate to severe abnormal EEG findings was also high (100% and 87.5%, respectively). Patients with severe disease and poor prognosis had slow waves and δ waves.

Immunotherapy is the main treatment for anti-NMDAR encephalitis. Administration of immunotherapy at first onset reduces the risk of relapses [5]. However, there is no standard treatment for relapse at present, and the treatment principle for first-onset anti-NMDAR encephalitis is followed. Immunotherapy includes first- and second-line and chronic immunotherapy. First-line immunotherapy, including glucocorticoid and intravenous immunoglobulin administration and plasma exchange, is preferred. Patients with involuntary movements, consciousness disturbance, central hypoventilation, and accompanying hypoalbuminemia and pulmonary infection may respond poorly to first-line treatments. Admission to the ICU, intracranial hypertension, and increased neutrophil-to-lymphocyte ratio might be significant factors affecting the response to first-line treatments [15]. Second-line immunotherapy can be administered when first-line immunotherapy is ineffective. This study showed that 60% and 40% of the patients at first onset and relapse, respectively, were treated with two or more types of first-line immunotherapy. Administration of a combination of multiple immunotherapies is not commonly noted. Second-line immunotherapy should be administered to patients with MRS scores ≥ 3 and with failure of first-line immunotherapy for 2 weeks [20]. After active immunotherapy, the QOL of the patients with first-onset and relapsing anti-NMDAR encephalitis significantly improved (*P* < 0.000). Active immunotherapy remains effective at relapse. This study reported that all patients relapsed after discontinuing immunotherapy (relapse time after discharge, 18.3 ± 16.5; duration of medication use, 3.10 ± 2.69 months; *P* = 0.022). Anti-NMDAR encephalitis often relapses during the process of termination of immunotherapy or decreasing low-dose corticosteroid [10]. Chronic immunotherapy generally comprises administration of oral azathioprine or mycophenolate for at least 1 year, which was recommended by Dalmau et al. [9] Chronic immunotherapy is necessary in all cases, except for patients with mild symptoms at first onset and for those who underwent teratoma removal [9, 21], suggesting that chronic immunotherapy is appropriate for most cases. These notions need to be supported by evidence from multicenter studies with large sample populations and longer-term follow-up. Further, the selection of immunotherapy should be based on the characteristics and requirements of each case.

The duration of taking AEDs was < 1 year (median 0.5 years) after first discharge, but the relapse rate of epilepsy was low. After 2 years of follow-up, patients with epileptic seizures in the acute stage did not experience a repeat of epileptic seizures [22]. There was no significant difference in the final prognosis of epilepsy between the 3- and > 3-month AED therapy groups [22]. The gradual recovery from encephalitis can decrease the number and duration of seizures, suggesting that patients with first-onset and relapsing anti-NMDAR encephalitis with concomitant epilepsy do not need long-term AED intake.

Anti-NMDAR encephalitis can relapse once or multiple times, with an average relapse interval of 5 months [1, 23]. In this study, the average relapse time was 18.3 months; however, it significantly differed among the patients. This may be related to differences in the patients’ conditions and immunotherapy after discharge. Patients with negative tumor screening results [9], those with serious first-onset illness, those who do not receive immunotherapy at first onset [5], those with no second-line immunotherapy, and those treated with nonstandard immunotherapy are at a high risk for relapse [1, 23, 24]. Early aggressive immunotherapy (and tumor removal, if tumor is detected) appears to reverse the disease [25], preventing relapses and disability in anti-NMDAR encephalitis. Currently, there is no consensus regarding consolidation or maintenance immunotherapy in remission. Standardized immunotherapy is the key to preventing relapse, and all relapse patients are recommended to receive long-term immunotherapy.

The average treatment cycle of patients with severe anti-NMDAR encephalitis is 1–2 months in the ICU; the mortality rate is 2.9–9.5%, while a few patients may show full recovery after > 2 years [1, 23, 26]. There is a lack of studies with large sample populations reporting on the long-term prognosis of patients with anti-NMDAR encephalitis. Patients who receive immunotherapy early and those who do not have severe symptoms have better prognosis [3]. MRI abnormalities and cognitive impairment have been reported to be significant predictors of poor short-term prognosis [27], but contradictory findings exist [28]. Further, admission to the pediatric ICUs and consciousness disturbance are also related to prognosis [20]. The level of NLR family pyrin domain containing the 3-inflammasome (NLRP3) in the CSF could represent the severity of anti-NMDAR encephalitis, and the decrease in the CSF levels of NLRP3 inflammasome could serve as an indicator for the prognosis of this disease [29]. Remarkable differences in prognosis may occur in different populations. Factors affecting prognosis must be confirmed by large-scale multicenter prospective studies in the future. Self-controlled studies on anti-NMDAR encephalitis are rare. This study makes a significant contribution to the literature because the results characterize relapsing anti-NMDAR encephalitis. This information will aid in disease diagnosis and treatment as well as in the prevention of relapse. The limitation of this study is that we only evaluated first-onset and initial relapse cases. Patients with anti-NMDAR encephalitis can relapse many times. As this study aimed to compare the differences between first onset of the disease and relapse, we did not track or evaluate cases of multiple relapses. Patients with many relapses constitute an interesting group for future study, particularly, to ascertain the reasons for the relapse.

In conclusion, Patients have less severe symptoms and better QOL at relapse than at first onset. The rate of antibody positivity in the CSF was higher at both first onset and relapse, and increasing antibody titers have implications for relapse. The incidence of abnormal EEG findings was high; however, the specificity of the routine examination of the CSF and brain MRI was low. Active immunotherapy can significantly improve the QOL at both first onset and relapse. Premature termination of immunotherapy may be an important cause of relapse; however, prolonged AED intake is unnecessary. A long-term follow-up study on relapse will help to determine the reasons for relapse and improve diagnosis and treatment.

## Data Availability

The datasets generated during and/or analyzed during the current study are available from the corresponding author on reasonable request.
